# Organizational value realization from AI-enabled digital health technologies in long-term care: roles of human–AI collaboration and digital literacy

**DOI:** 10.3389/fmed.2026.1958359

**Published:** 2026-10-14

**Authors:** Lin-Yu Tsai, Yi-Nuo Shih, Szu-Kou Lee, Mingchih Chen, Yi-Chen Lan

**Affiliations:** 1 Graduate Institute of Business Administration, Fu Jen Catholic University, New Taipei City, Taiwan; 2 MS Program in Transdisciplinary Long-Term Care, College of Medicine, Fu Jen Catholic University, New Taipei City, Taiwan; 3 Department of Occupational Therapy, College of Medicine, Fu Jen Catholic University, New Taipei City, Taiwan; 4 Artificial Intelligence Development Center, Fu Jen Catholic University, New Taipei City, Taiwan; 5 Graduate Institute of Applied Science and Engineering, Fu Jen Catholic University, New Taipei City, Taiwan

**Keywords:** artificial intelligence, digital health technologies, digital literacy, healthcare management, human–AI collaboration, information systems success model, long-term care, value realization

## Abstract

Rapid population aging has intensified pressures on long-term care (LTC) systems, exacerbating workforce shortages and challenges to sustainable healthcare delivery. Although AI-enabled digital health technologies (DHTs) have emerged as promising tools to address these challenges, existing research has focused largely on technology adoption and system use in hospital settings, providing limited understanding of how AI-enabled DHTs realize organizational value and optimize healthcare delivery in LTC organizations. This study aimed to develop and examine an integrated framework for understanding organizational value realization from AI-enabled DHTs in LTC organizations, focusing on the interrelated roles of technological quality, human–AI collaboration, and workforce digital capability. The study also sought to generate evidence to inform healthcare management strategies for optimizing healthcare delivery. A cross-sectional survey was conducted among 257 staff from multiple LTC facilities in Taiwan who had used AI-enabled DHTs for at least six months. Data were analyzed using partial least squares structural equation modeling (PLS-SEM) and importance-performance map analysis (IPMA). The proposed model explained 77.9% of the variance in perceived organizational value, together with substantial variance in user satisfaction (R^2^ = 0.763) and system use (R^2^ = 0.652). Human–AI collaboration quality showed the strongest positive association with user satisfaction and was positively associated with system use. User satisfaction was more strongly associated with perceived organizational value than system use. Digital literacy showed significant but differing moderation effects across these relationships. IPMA identified AI explainability, adaptive personalization, system stability, and functional completeness as priority areas for improvement. These findings advance understanding of organizational value realization from AI-enabled DHTs in LTC organizations by highlighting the roles of human–AI collaboration and digital literacy. They underscore the value of explainable, adaptive, and human-centered AI design in supporting positive user experiences with AI-enabled DHTs. Workforce digital literacy may further shape how these technology experiences relate to perceived organizational value. These insights provide evidence to inform healthcare management strategies that align AI system design and workforce development with organizational objectives to support healthcare delivery optimization and sustainable long-term care in aging societies.

## Introduction

Many countries are rapidly transitioning into super-aged societies, where increasing longevity is accompanied by a growing prevalence of chronic conditions, multimorbidity, and functional decline. As a result, long-term care (LTC) systems play an increasingly important role in maintaining health, independence, and quality of life among older adults (1–3). To address rising care demands and persistent workforce shortages, LTC organizations have increasingly adopted digital health technologies (DHTs), including artificial intelligence (AI)-enabled DHTs that support care coordination, monitoring, documentation, and clinical decision making.

In this study, digital health technologies (DHTs) are defined in accordance with the World Health Organization framework as digital systems and applications designed to support health and care processes, including decision support, workflow coordination, and service delivery (3). Common applications in LTC settings include electronic health records, telemedicine platforms, wearable monitoring devices, and environmental sensing technologies (4–6). AI-enabled DHTs refer to DHTs that incorporate artificial intelligence (AI) capabilities to support functions such as data analysis, anomaly detection, prediction, recommendations, personalized care, workflow automation, and clinical or care-related decision support (7–9). These AI capabilities may be embedded within broader digital systems rather than presented to users as standalone AI applications. Accordingly, AI-enabled DHTs encompass heterogeneous digital systems through which AI capabilities are integrated into care delivery and management. Collectively, AI-enabled DHTs have the potential to improve care quality, operational efficiency, care coordination, and service sustainability, making them an increasingly important component of digital transformation in LTC (7–10).

Despite their considerable potential, healthcare organizations frequently encounter difficulties in realizing meaningful organizational value from AI-enabled DHTs. In this study, organizational value refers to staff perceptions of the value realized from AI-enabled DHTs across four organizationally relevant domains: care quality, operational efficiency, resource utilization, and workforce capability development. The adoption of AI-enabled DHTs alone does not automatically translate into sustained improvements in care delivery or organizational performance. Instead, value realization depends on how effectively AI-enabled DHTs are embedded within routine care delivery, integrated into organizational workflows, and aligned with the broader organizational context in which they are implemented (8–10). These implementation challenges suggest that understanding organizational value realization requires theoretical frameworks capable of explaining how system characteristics, user experiences, and organizational outcomes are connected throughout the value realization process.

Among the theoretical frameworks used to evaluate digital technologies, the Technology Acceptance Model (TAM) and the DeLone and McLean (D&M) Information Systems Success Model have been the most widely applied. While TAM primarily explains technology adoption through perceived usefulness and perceived ease of use (11), the D&M model provides a more comprehensive framework for explaining how system characteristics influence system use, user satisfaction, and ultimately organizational value (i.e., net benefits in the D&M model) (12, 13). Because it evaluates information systems beyond simple adoption outcomes, the D&M model has been widely applied in healthcare and digital health research. However, these frameworks were largely developed when information systems functioned primarily as passive information-processing tools. As AI systems embedded within AI-enabled DHTs increasingly collaborate with healthcare professionals in decision making, traditional information systems success models may not fully explain how organizational value is realized in AI-enabled care environments (14, 15).

Human–AI collaboration has therefore emerged as an increasingly important consideration in AI-enabled healthcare. Unlike conventional information systems that primarily provide information or support predefined tasks, AI systems embedded within AI-enabled DHTs may provide predictive, recommendation, adaptive, or other decision-support functions that can support users’ work and care-related decision processes (16–19). This expands the user–system relationship beyond interaction with a digital tool toward forms of collaboration in which AI capabilities may contribute to interpretation and decision support. Consequently, understanding the use of AI-enabled DHTs requires consideration not only of the technical quality of the technology or the information it provides, but also of the quality of users’ collaborative interaction with AI systems.

Human–AI collaboration quality can therefore be conceptualized as the overall quality of the collaborative relationship between users and AI systems in work and decision processes. This interactional quality is theoretically distinct from the established D&M quality dimensions. Information quality concerns the characteristics of system outputs, system quality concerns the technical and functional performance of the system, and service quality concerns the support provided to users (12, 13). Human–AI collaboration quality instead concerns whether interaction with AI enables users to understand AI-generated outputs, retain appropriate professional judgment, receive support responsive to their needs and context, and interact with the system under appropriate safeguards (14, 15, 18, 20, 21). Although these characteristics represent conceptually distinct aspects of human–AI interaction, they converge theoretically on a common question: whether the AI system enables an effective and appropriate collaborative relationship with the human user. Human–AI collaboration quality therefore captures an interactional property of AI-enabled DHTs that is not fully represented by conventional assessments of information, system, or service quality.

This distinction has implications for information systems success. When users perceive AI-related functions as understandable, supportive of professional judgment, responsive to their needs and context, and appropriately safeguarded, they may be more willing and able to incorporate AI-enabled DHTs into routine work and may evaluate their experience with these technologies more positively. Human–AI collaboration quality therefore extends the D&M model by introducing an interactional mechanism through which the quality of users’ collaboration with AI systems is theoretically expected to be associated with their use of and satisfaction with AI-enabled DHTs. Extending the D&M model in this way shifts the theoretical focus from evaluating the digital technology alone toward considering the quality of the relationship through which users engage with AI capabilities.

In addition to collaborative system characteristics, digital literacy has become increasingly important in determining whether healthcare organizations can successfully realize value from digital technologies. Digital literacy influences not only how healthcare professionals operate digital systems but also how effectively they interpret information, integrate technology into care processes, and translate system use into meaningful organizational outcomes. This issue is particularly relevant in LTC settings, where care staff often possess diverse educational backgrounds, professional responsibilities, and levels of digital experience. Previous studies suggest that digital literacy facilitates effective engagement with digital technologies and contributes to improved care delivery outcomes (8, 22, 23). Accordingly, digital literacy may function not only as an antecedent of technology use but also as an organizational workforce capability that shapes how technology engagement is associated with value realization from AI-enabled DHTs.

Although previous studies have identified important determinants of digital health implementation and information systems success, existing evidence provides only a partial theoretical explanation of organizational value associated with AI-enabled DHTs in LTC. The D&M model primarily explains value realization through relationships among information, system, and service quality, system use, user satisfaction, and net benefits. However, this logic does not explicitly account for the quality of users’ collaborative interaction with AI systems, nor does it fully address whether the relationships of technology use and satisfaction with organizational value may vary according to users’ digital literacy. Beyond these theoretical limitations, existing research has often emphasized proximal or task-specific outcomes, with less attention to broader organizational value encompassing care quality, operational efficiency, resource utilization, and workforce capability development (24–30). Examining these broader dimensions provides a more comprehensive perspective on organizational value in healthcare digital transformation (31–33). Furthermore, existing evidence remains concentrated in hospital settings or single-institution studies, limiting the applicability of existing knowledge to LTC environments, where workforce composition, care workflows, and organizational contexts differ substantially (24–27). Collectively, these limitations indicate the need for a framework that integrates traditional information systems quality, human–AI collaboration, and workforce digital literacy in examining perceived organizational value from AI-enabled DHTs in LTC.

To address these theoretical and empirical gaps, this study investigates factors associated with perceived organizational value realization from AI-enabled DHTs in LTC organizations. Grounded in the D&M model, the proposed framework incorporates human–AI collaboration quality as an interactional quality construct relevant to users’ engagement with embedded AI systems and digital literacy as a workforce capability that may shape value realization. Specifically, the study examines the interrelationships among technological quality, human–AI collaboration quality, system use, user satisfaction, digital literacy, and perceived organizational value. Through this framework, this study seeks to contribute to the literature on AI-enabled digital health implementation in three ways: (1) advancing understanding of value realization in AI-enabled care environments; (2) extending the D&M model by conceptualizing and empirically examining human–AI collaboration quality as a distinct dimension of information systems success; and (3) broadening the theoretical role of digital literacy beyond technology adoption and use by conceptualizing it as a workforce capability that may serve as a boundary condition in organizational value realization from AI-enabled DHTs.

## Methods

### Study design and research model

This study adopted a cross-sectional survey design involving staff from long-term care (LTC) organizations in Taiwan with experience using AI-enabled digital health technologies (DHTs). Taiwan provides a relevant setting for this study because it is among the world's fastest-aging societies and has actively promoted digital transformation within its long-term care system.

For the purpose of the survey, AI-enabled DHTs were operationalized as a heterogeneous range of smart technology systems used in LTC settings. In the participant-facing Chinese questionnaire, the term 「智慧科技系統」 (“smart technology systems”) was used to represent AI-enabled DHTs. At questionnaire commencement, participants were provided with a visual illustration defining the scope of these technologies, encompassing electronic health records, health-monitoring devices, data-driven decision analysis and recommendations, anomaly-detection and alert systems, and integrated smart-care management platforms (Supplementary Figure S1; original Chinese version in Supplementary File S1, p. 2). The illustration provided respondents with a common frame of reference for the heterogeneous AI-enabled DHTs examined in this study rather than restricting their responses to a specific technology, product, or AI function.

The AI-specific context was further reinforced in the human–AI collaboration quality section of the questionnaire, which explicitly addressed users’ interaction and collaborative relationship with AI systems (Supplementary File S1, p. 8). The research model was grounded in the DeLone and McLean (D&M) Information Systems Success Model, which conceptualizes information quality, system quality, and service quality as core antecedents of use, user satisfaction, and net benefits. Building on this framework, this study extends the D&M model by incorporating human–AI collaboration quality as an additional quality dimension. This construct captures interactional and collaborative characteristics of AI systems embedded within AI-enabled DHTs that are not represented in the traditional information, system, and service quality constructs, drawing on concepts from the human-centered AI and human–AI collaboration literature.

Consistent with the study objective, the D&M model's net benefits construct was operationalized as users’ perceived organizational value using four dimensions of the Balanced Scorecard (BSC): care quality, operational efficiency, resource utilization, and capability development. These dimensions reflect medium- to long-term care-related value outcomes. Within the model, use and user satisfaction were specified as intermediate constructs linking upstream quality factors to net benefits and representing the value realization pathway of AI-enabled DHT implementation. Digital literacy was incorporated as a moderating variable to examine its role as a boundary condition shaping the strength of the relationships between these intermediate constructs and organizational-level outcomes. Figure 1 illustrates the proposed research model, depicting the hypothesized associations among system characteristics, user experiences, and organizational value, with digital literacy modeled as a moderator.

**Figure 1:**
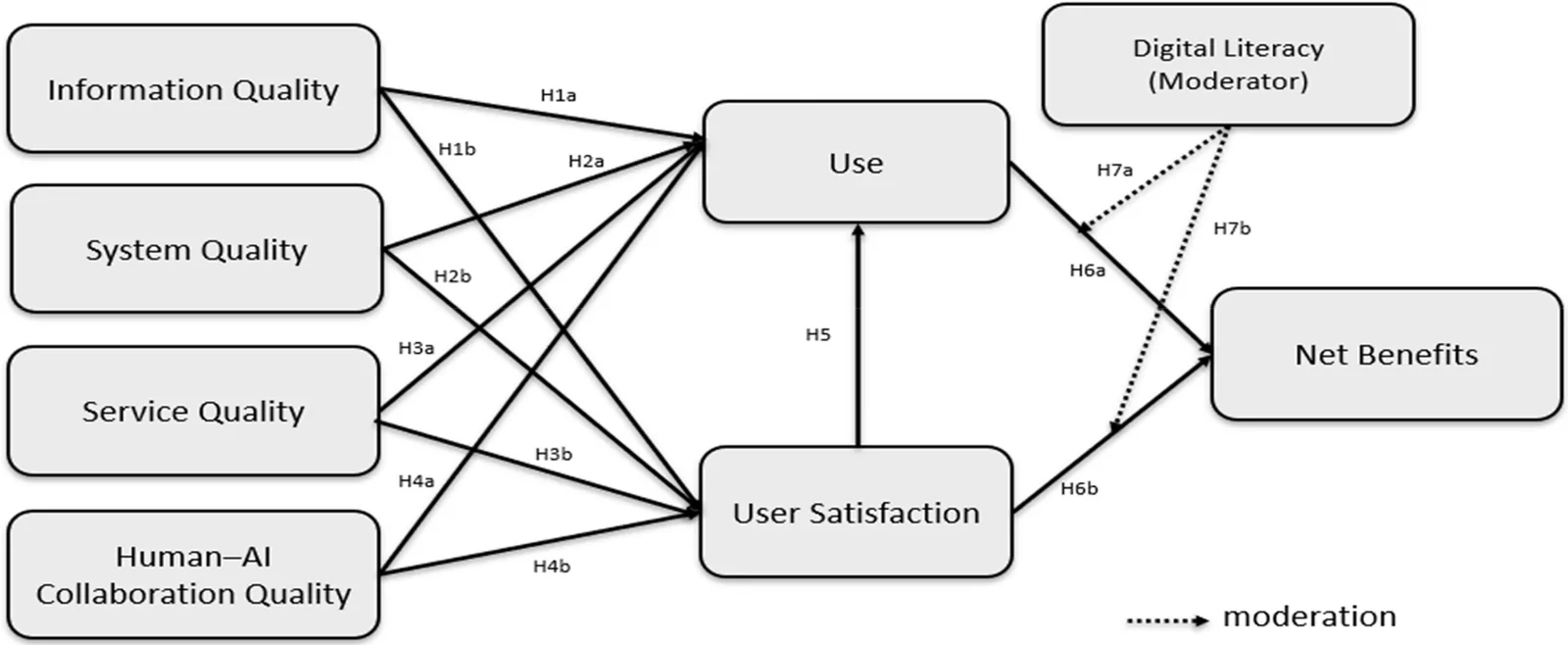
Proposed research model. The model illustrates the hypothesized relationships among information quality, system quality, service quality, human–AI collaboration quality, use, user satisfaction, and net benefits within the DeLone and McLean framework, with digital literacy modeled as a moderator.

### Hypotheses

According to the D&M model, information quality, system quality, and service quality are core quality dimensions associated with system use and user satisfaction (13, 40). Previous studies in digital health and health information systems have consistently supported these relationships across a range of healthcare settings (24, 25). Based on this theoretical foundation, the following hypotheses were developed:

H1a: Information quality is positively associated with use.

H1b: Information quality is positively associated with user satisfaction.

H2a: System quality is positively associated with use.

H2b: System quality is positively associated with user satisfaction.

H3a: Service quality is positively associated with use.

H3b: Service quality is positively associated with user satisfaction.

As AI systems embedded within AI-enabled DHTs increasingly provide predictive, adaptive, explanatory, or other decision-support functions, the quality of users’ interaction with these systems may be relevant to how effectively human and AI capabilities are combined in practice (19, 35). Users may be more willing to engage with AI-enabled DHTs when the embedded AI systems support rather than replace human judgment, respond to individual needs, provide understandable AI-related functions, and appropriately safeguard personal information (17, 36, 41). Moreover, characteristics of human–AI interaction such as explainability and effective communication may shape users’ trust in and evaluations of their interactions with AI systems (18, 19). Human–AI collaboration quality is therefore theoretically expected to be positively associated with both use and user satisfaction. Accordingly, the following hypotheses were developed:

H4a: Human–AI collaboration quality is positively associated with use.

H4b: Human–AI collaboration quality is positively associated with user satisfaction.

Within the D&M model, use and user satisfaction are closely related constructs that jointly contribute to organizational value (i.e., net benefits) (13). In healthcare settings where system use is mandatory or semi-mandatory, usage frequency alone may not adequately reflect meaningful engagement with digital technologies. Instead, user satisfaction has been shown to better capture users’ overall evaluation of system performance and to be associated with continued and effective system use (26, 37, 42). These considerations are particularly relevant in LTC settings, where digital systems are routinely used for documentation and care coordination. Accordingly, the following hypothesis was developed:

H5: User satisfaction is positively associated with use.

The D&M model further proposes that both system use and user satisfaction contribute to organizational value (i.e., net benefits) (13). Although previous studies generally support these relationships, evidence suggests that user satisfaction is often more consistently associated with organizational outcomes than system use across different healthcare contexts (24, 25, 43). Therefore, the following hypotheses were developed:

H6a: Use is positively associated with net benefits.

H6b: User satisfaction is positively associated with net benefits.

In this study, digital literacy refers to users’ perceived capability to apply digital information, communicate and participate through digital technologies, and address digital work-related tasks or problems. Previous research has identified digital literacy and related digital capabilities as important workforce resources associated with engagement in digital health, effective technology use, and digital transformation in healthcare organizations (22, 23, 44–46).

System use represents engagement with the technology, whereas user satisfaction reflects users’ evaluation of that experience; neither construct alone captures users’ capability to apply digital technologies effectively to work-related tasks and problems. Workplace and healthcare research further suggests that digital literacy and related digital capabilities influence users’ ability to integrate digital technologies into work practices and respond effectively to technology-enabled work requirements (47–49). Accordingly, comparable levels of system use or user satisfaction may be associated with different levels of organizational value depending on users’ capability to apply digital technologies effectively. However, existing evidence provides limited basis for specifying the direction of these moderation effects. Digital literacy may therefore function as a workforce capability that conditions how system use and user satisfaction are associated with organizational value. Accordingly, the following non-directional moderation hypotheses were developed:

H7a: Digital literacy moderates the association between use and net benefits.

H7b: Digital literacy moderates the association between user satisfaction and net benefits.

### Participants and data collection

A convenience sampling approach was employed using a targeted, closed online recruitment strategy to recruit staff from LTC organizations. The sampling approach aimed to capture diverse LTC settings and enhance the generalizability of findings across care environments. Study information and the survey link were distributed to current staff at participating LTC facilities by designated institutional contacts through internal LINE groups (group chats on the LINE instant-messaging platform) or mailing lists. The survey was not publicly accessible. Eligibility criteria were: (1) age 20–75 years; (2) current employment in an LTC organization; (3) at least 6 months of experience using AI-enabled DHTs as operationally defined and illustrated in the questionnaire, including experience accumulated in previous employment; and (4) provision of informed consent.

The questionnaire was administered using Google Forms. Upon accessing the form, participants were first required to provide electronic informed consent by confirming that they had read the study information and agreed to participate; only those who selected “Yes, I agree” could continue. Eligibility screening was used to ensure that only eligible participants could proceed. Google Forms settings limited responses to one submission per participant and recorded timestamps for quality control. Data collection was conducted between October 8 and November 14, 2025. A total of 273 responses were received, and after excluding ineligible or incomplete submissions, 257 valid responses were retained for analysis.

To enhance external validity, recruitment included multiple LTC facilities. Sample size adequacy was evaluated based on established PLS-SEM guidance and the complexity of the proposed model (50, 51)**.** The achieved sample size (*N* = 257) exceeded conventional minimum requirements for model estimation. A *post hoc* assessment based on the observed structural path coefficients further indicated that the sample met the estimated sample-size requirements for most of the observed direct effects.

### Ethics considerations

This study was approved by the Human Research Ethics Committee of Fu Jen Catholic University (FJU-JRB No. C114027). Participation was voluntary, and electronic informed consent was obtained from all participants prior to survey completion.

### Research variables and measurement instruments

Based on the proposed framework, eight latent constructs were measured: system quality, information quality, service quality, human–AI collaboration quality, use, user satisfaction, digital literacy, and net benefits. All constructs were assessed using a 7-point Likert scale (1 = strongly disagree to 7 = strongly agree). As described above, the measurement items were administered within the participant-facing context of AI-enabled DHTs established at questionnaire commencement, with the human–AI collaboration section explicitly referring to users’ interaction and collaboration with AI systems (Supplementary Figure S1; Supplementary File S1, pp. 2 and 8). Table 1 presents the definitions, measurement items, and corresponding literature sources for all study constructs.

**Table 1:** Definitions of study constructs and corresponding self-reported measurement items, assessed using a 7-point Likert scale.

Construct	Definition	Measurement Items	Source
Information Quality (INF)	Users’ perceived quality of the information produced by the system, including accuracy, task relevance, and clarity.	INF1: The system records and provides information that meets care-work needs. INF2: The information/content provided by the system is accurate. INF3: The information provided by the system is clear and easy to understand.	(24, 34)
System Quality (SYS)	Users’ perceived technical and functional quality of the system itself, including stability, functional completeness, and interface clarity.	SYS1: The system operates very stably. SYS2: The system has complete functions that are helpful for my work. SYS3: The user interface is well-organized and easy to learn.	(25, 26)
Service Quality (SER)	Users’ perceived quality of supporting services related to the system, including reliability, responsiveness, empathy, and professional support.	SER1: Support staff provide reliable service. SER2: Support staff understand my needs. SER3: Support staff respond to my issues in a timely manner. SER4: Support staff provide sufficient user guidance and training.	(25, 26)
Human–AI Collaboration Quality (HAI quality)	Users’ overall perceived quality of collaborative interaction with AI systems during task execution, reflected through support for human judgment, explainability, adaptive personalization, and safeguards for personal information.	HAI1: The system assists my work rather than replacing my judgment or decisions. HAI2: The logic behind the system's recommendations or predictions is easy to understand. HAI3: The system can adjust how information or content is presented based on user needs or feedback. HAI4: The system provides mechanisms such as passwords and access control to protect personal data and prevent leakage.	(16, 35, 36)
Use (USE)	The proactivity and frequency with which users employ the system in their work context.	USE1: I often use the system to complete my daily work. USE2: I consider this system essential for accomplishing my tasks. USE3: I proactively use this system when a task needs to be completed.	(25, 26, 37)
User Satisfaction (SAT)	Users’ affective and cognitive evaluations of the system's overall performance based on usage experience.	SAT1: I am satisfied with the overall service provided by the system. SAT2: My experience using the system is pleasant. SAT3: I am willing to recommend this system to others.	(26, 34)
Digital Literacy (DIG)	Users’ perceived capability to apply digital information, communicate and participate through digital technologies, and address digital work-related tasks or problems.	DIG1: Based on the information displayed by the system, I can take appropriate care actions or work decisions. DIG2: I can use digital platforms (e.g., LINE, email) to communicate clearly with colleagues or support staff. DIG3: I can input care-related data, feedback, or suggestions within the system. DIG4: When encountering difficulties operating the system, I can try to solve them on my own or seek help.	(38, 52–54)
Net Benefits (NB)	Users’ overall perception of value realized from the system in care delivery, reflecting its contributions to care quality, operational efficiency, resource utilization, and capability development.	NB1: The system helps improve client safety and quality of care. NB2: The system saves organizational resources (e.g., paper, utilities, medical supplies, workforce). NB3: The system improves the efficiency of service work. NB4: System-related training enhances my learning efficiency and professional skills.	(31, 33, 39)

Human–AI collaboration quality was developed from prior literature on human-centered and human–AI interaction and conceptualized as an overarching perceptual construct reflecting users’ overall evaluation of the quality of collaborative interaction with AI systems. Four characteristics relevant to effective human–AI interaction in AI-enabled DHTs were operationalized, namely explainability, support for human judgment, adaptive personalization, and safeguards for personal information. These characteristics capture complementary aspects of a common interactional domain and collectively reflect users’ overall evaluation of the quality of collaboration with AI systems. Support for human judgment reflects whether AI complements rather than replaces human judgment (17); explainability concerns whether AI-related functions are understandable to users (36); adaptive personalization reflects responsiveness to users’ needs and context (20); and safeguards for personal information concern the protection of sensitive information during technology use (41). Collectively, these characteristics reflect whether users perceive their interaction with AI systems as supportive, understandable, responsive, and appropriately safeguarded. Accordingly, the four items were conceptualized as reflective manifestations of a single underlying perception of human–AI collaboration quality rather than as separate latent constructs.

Net benefits were developed with reference to the Balanced Scorecard framework and operationalized across four organizational value domains: care quality, operational efficiency, resource utilization, and workforce capability development. The remaining constructs were adapted from established instruments and refined for the LTC context.

The draft questionnaire was then reviewed by an eight-member expert panel with relevant expertise and experience in long-term care, digital health, and information systems. Through group discussion, the panel assessed item relevance, contextual appropriateness, wording clarity, and questionnaire structure. Based on the expert review, questionnaire items were refined to improve clarity and contextual relevance for LTC respondents.

Following these revisions, the questionnaire was pilot tested with 27 LTC participants to assess item clarity, comprehensibility, survey completion time, and preliminary measurement properties. The pilot results showed satisfactory preliminary reliability and convergent validity across the measurement constructs and supported use of the questionnaire for formal data collection. No further substantive changes to the measurement items were required after pilot testing.

### Data analysis

Data were analyzed using partial least squares structural equation modeling (PLS-SEM) with SmartPLS (version 4.1.1.4, SmartPLS GmbH) (55). PLS-SEM was selected because this study extends an established theoretical framework by incorporating newly conceptualized constructs and moderation effects, while simultaneously examining multiple interrelated relationships. In addition, PLS-SEM is appropriate for prediction-oriented research and is robust to non-normal data distributions, making it well suited for analyzing survey data collected from heterogeneous LTC settings (50, 56, 57). The measurement model was evaluated by assessing indicator reliability, internal consistency reliability (Cronbach's *α*, rho_A, and composite reliability), convergent validity (average variance extracted), and discriminant validity using the heterotrait–monotrait ratio (HTMT) and Fornell–Larcker criterion (57, 58). Indicator loading ≥0.70 and internal consistency reliability values ≥0.70 and average variance extracted (AVE) values ≥0.50 were considered acceptable, while HTMT values <0.90 were used as the primary criterion for discriminant validity (57, 58). The structural model was assessed using bootstrapping with 5,000 resamples to evaluate path coefficients and significance levels. Model explanatory power and effect sizes were examined using R^2^ and f^2^, respectively, and model fit was assessed using the standardized root mean square residual (SRMR). Variance inflation factors (VIFs) were used to assess multicollinearity. Moderation effects were tested using the product indicator approach, and simple slope analyses were conducted to interpret significant interaction effects (59, 60). In addition, importance–performance map analysis (IPMA) was conducted to further identify the most important and influential constructs and their associated measurement items, allowing for the prioritization of areas for improvement within the model (56, 57). IPMA complements conventional PLS-SEM by translating statistical relationships into implementation priorities, thereby identifying areas where improvements are most likely to enhance organizational value realization. The target construct was selected based on its role as the primary indicator of value realization in care delivery within the structural model.

## Results

### Participant characteristics

A total of 257 participants were included in the analysis. The sample was predominantly female (82.9%), with males accounting for 16.7% of participants; a small proportion (0.4%) of responses were categorized as other. The largest age group was 41–50 years (31.9%), followed by 31–40 years (21.8%) and 51–60 years (16.7%). Most participants held a university degree (45.1%), followed by junior college qualifications (23.4%) and a high school education or below (20.2%); 11.3% had a master's degree.

Regarding work experience, the majority of participants reported 3–10 years of service (33.9%), followed by 1–3 years (30.4%). Participants were primarily employed in day care centers (36.2%) and residential LTC facilities (37.3%), with 25.3% working in home-based care services.

Job roles were classified into mutually exclusive categories for reporting and analysis purposes. Care assistants constituted the largest group (57.6%), followed by nurses (14.8%), managerial staff (e.g., supervisors, directors, or operations leads; 13.6%), social workers (3.9%), professional staff with concurrent managerial roles (5.0%), and other roles (5.1%).

A detailed breakdown of participant demographic characteristics and facility types is presented in Table 2. The sample reflects a broad representation of long-term care (LTC) staff roles and care settings, supporting the relevance of the findings to real-world care delivery contexts.

**Table 2:** Demographics of the participants (*N* = 257).

Characteristics	Count	% ^a^	Cumulative %^b^
Sex			
Male	43	16.7	16.7
Female	213	82.9	99.6
Other	1	0.4	100.0
Age			
≤30 years	47	18.3	18.3
31–40 years	56	21.8	40.1
41–50 years	82	31.9	72.0
51–60 years	43	16.7	88.7
≥61 years	29	11.3	100.0
Education			
High school (vocational) or below	52	20.2	20.2
Junior college/diploma	60	23.4	43.6
Bachelor's degree	116	45.1	88.7
Master's degree	29	11.3	100.0
Tenure (current organization)			
≤6 months	23	8.9	8.9
>6 to 12 months	25	9.7	18.7
>1 to 3 years	78	30.4	49.0
>3 to 10 years	87	33.9	82.9
>10 years	44	17.1	100.0
Type of care organization			
Home-based care services	65	25.3	25.3
Adult day-care center	93	36.2	61.5
Residential long-term care facility	96	37.3	98.8
Other	3	1.2	100.0
Job role			
Care assistant	148	57.6	57.6
Nurse	38	14.8	72.4
Social worker	10	3.9	76.3
Professional staff with concurrent managerial role	13	5.0	81.3
Managerial staff (e.g., supervisor, director, operations lead)	35	13.6	94.9
Other	13	5.1	100.0

a % denotes the proportion within the full sample.

b Cumulative % is the running total within each characteristic category.

### Measurement model evaluation

The measurement model demonstrated satisfactory reliability and validity (Tables 3–5) (57). All indicator loadings exceeded 0.70, supporting indicator reliability. Internal consistency reliability was satisfactory, with Cronbach's *α*, rho_A, and composite reliability (CR) values exceeding 0.70 for all constructs. Convergent validity was also supported, as all average variance extracted (AVE) values exceeded the recommended threshold of 0.50 (57).

**Table 3:** Construct reliability and validity.

Construct	Cronbach's *α*	rho_A	CR	AVE
Information Quality	0.905	0.907	0.940	0.840
System Quality	0.913	0.914	0.946	0.853
Service Quality	0.938	0.939	0.956	0.843
Human–AI Collaboration Quality	0.905	0.912	0.934	0.780
Use	0.956	0.958	0.972	0.919
User Satisfaction	0.952	0.952	0.969	0.912
Digital Literacy	0.927	0.929	0.948	0.820
Net Benefits	0.939	0.942	0.956	0.846

rho_A, Dijkstra-Henseler's rho_A; CR, composite reliability; AVE, average variance extracted.

**Table 4:** HTMT discriminant validity^a^ (heterotrait–monotrait ratio).

Construct	HAI Quality	Use	User Satisfaction	Service Quality	Net Benefits	Digital Literacy	System Quality	Information Quality
HAI Quality								
Use	0.759	- ^b^	- ^b^	- ^b^	- ^b^	- ^b^	- ^b^	- ^b^
User Satisfaction	0.892	0.816	- ^b^	- ^b^	- ^b^	- ^b^	- ^b^	- ^b^
Service Quality	0.855	0.619	0.862	- ^b^	- ^b^	- ^b^	- ^b^	- ^b^
Net Benefits	0.845	0.797	0.890	0.737	- ^b^	- ^b^	- ^b^	- ^b^
Digital Literacy	0.853	0.874	0.880	0.721	0.896	- ^b^	- ^b^	- ^b^
System Quality	0.881	0.694	0.890	0.942	0.795	0.805	- ^b^	- ^b^
Information Quality	0.837	0.720	0.783	0.806	0.710	0.767	0.834	- ^b^

HAI, human–AI collaboration.

a HTMT values below 0.85–0.90 typically indicate adequate discriminant validity; diagonal is blank by definition.

b Not applicable.

**Table 5:** Fornell–Larcker discriminant validity.^a^

Construct	HAI Quality	Use	User Satisfaction	Service Quality	Net Benefits	Digital Literacy	System Quality	Information Quality
HAI Quality	*0* *.* *883*	- ^b^	- ^b^	- ^b^	- ^b^	- ^b^	- ^b^	- ^b^
Use	0.725	*0*.*959*	- ^b^	- ^b^	- ^b^	- ^b^	- ^b^	- ^b^
User Satisfaction	0.785	0.798	*0*.*955*	- ^b^	- ^b^	- ^b^	- ^b^	- ^b^
Service Quality	0.759	0.588	0.779	*0*.*918*	- ^b^	- ^b^	- ^b^	- ^b^
Net Benefits	0.751	0.784	0.834	0.651	*0.920*	- ^b^	- ^b^	- ^b^
Digital Literacy	0.776	0.822	0.820	0.645	0.840	*0.906*	- ^b^	- ^b^
System Quality	0.781	0.655	0.795	0.861	0.713	0.715	*0.923*	- ^b^
Information Quality	0.764	0.674	0.698	0.747	0.640	0.679	0.751	*0.917*

HAI, human–AI collaboration; AVE, average variance extracted.

a Diagonal cells show the square root of AVE for each construct (italicized in journal layout). Off-diagonal cells are inter-construct correlations.

b Not applicable.

Discriminant validity was evaluated using both the heterotrait–monotrait ratio (HTMT) and the Fornell–Larcker criterion (57, 58). HTMT values were below the recommended threshold of 0.90 for most construct pairs. The HTMT value between system quality and service quality was 0.942, exceeding this threshold and indicating substantial overlap between the two constructs. Given this elevated value, additional assessment was conducted using bootstrapped HTMT inference. The 95% confidence interval ranged from 0.900 to 0.978, with the upper bound remaining below 1.00, providing additional evidence supporting the empirical distinction between the two constructs despite their substantial overlap. The Fornell–Larcker criterion was also satisfied for all constructs.

### Structural model evaluation

The structural model results are presented in Figure 2, and the corresponding hypothesis testing results are summarized in Table 6. Regarding system use, information quality (*β* = 0.249, *p* = .002) and human–AI collaboration quality (*β* = 0.196, *p* = .023) were positively associated with use. Service quality showed a significant negative association with use (*β* = −0.326, *p* = .002), opposite to the hypothesized direction, whereas system quality was not significantly associated with use (*β* = 0.045, *p* = .623).

**Figure 2:**
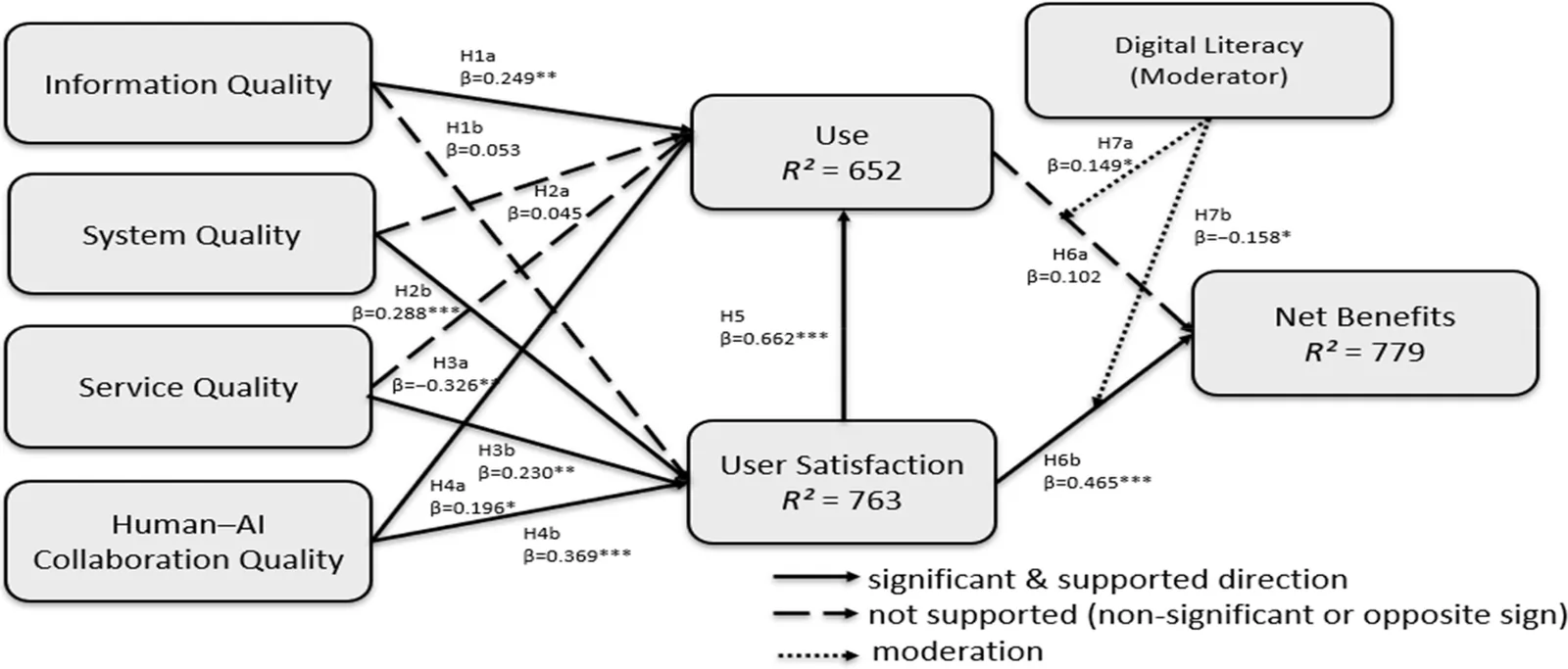
Structural model results. The figure presents the hypothesized relationships among the study constructs, including standardized path coefficients and coefficients of determination (R^2^) estimated using partial least squares structural equation modeling (PLS-SEM). Asterisks denote significance levels (* *p* < .05, ** *p* < .01, *** *p* < .001).

**Table 6:** Hypothesis testing results of the structural model (partial least squares structural equation modeling [PLS-SEM]).

Hypothesis & Path	*Β* ^a^	T-value^b^	*p*-value ^c^	Supported
H1a: Information quality → Use	0.249	3.105	0.002	Yes
H1b: Information quality → User Satisfaction	0.053	0.939	0.348	No
H2a: System quality → Use	0.045	0.492	0.623	No
H2b: System quality → User Satisfaction	0.288	3.665	<0.001	Yes
H3a: Service quality → Use	−0.326	3.123	0.002	No (−) ^d^
H3b: Service quality → User Satisfaction	0.230	3.200	0.001	Yes
H4a: Human–AI Collaboration Quality → Use	0.196	2.270	0.023	Yes
H4b: Human–AI Collaboration Quality → User Satisfaction	0.369	5.647	<0.001	Yes
H5: User Satisfaction → Use	0.662	8.412	<0.001	Yes
H6a: Use → Net Benefits	0.102	1.374	0.169	No
H6b: User Satisfaction → Net Benefits	0.465	8.123	<0.001	Yes
H7a: Digital Literacy×Use → Net Benefits	0.149	2.159	0.031	Yes
H7b: Digital Literacy×User Satisfaction → Net Benefits	−0.158	2.346	0.019	Yes

a β = standardized path coefficient.

b T-value from bootstrapping.

c *p*-value from bootstrapping.

d No (−) the estimated coefficient is significant but in the reverse direction of the hypothesized positive effect.

For user satisfaction, system quality (*β* = 0.288, *p* < .001), service quality (*β* = 0.230, *p* = .001), and human–AI collaboration quality (*β* = 0.369, *p* < .001) were positively associated with user satisfaction, whereas information quality was not significantly associated with user satisfaction (*β* = 0.053, *p* = .348).

User satisfaction was positively associated with both use (*β* = 0.662, *p* < .001) and net benefits (*β* = 0.465, *p* < .001). In contrast, the direct association between use and net benefits was not statistically significant (*β* = 0.102, *p* = .169).

The model demonstrated substantial explanatory power, explaining 65.2% of the variance in use (R^2^ = 0.652), 76.3% of the variance in user satisfaction (R^2^ = 0.763), and 77.9% of the variance in net benefits (R^2^ = 0.779). Approximate model fit was satisfactory, with standardized root mean square residual (SRMR) values of 0.044 for the saturated model and 0.061 for the estimated model, both below the recommended threshold of 0.08. Effect size (*f*^2^) analysis indicated effects of approximately 0.30 for the relationships between user satisfaction and use and between user satisfaction and net benefits, and an effect of approximately 0.17 for the relationship between human–AI collaboration quality and user satisfaction. Most remaining effects were small or negligible.

Collinearity diagnostics indicated that most variance inflation factors (VIFs) ranged from 2.72 to 4.99, suggesting no evidence of problematic multicollinearity among the predictor constructs. The interaction terms exhibited comparatively higher VIF values (9.34–10.12), a pattern commonly observed in moderation analyses involving product-indicator interaction terms, which are correlated with their constituent variables (56, 57, 59). Accordingly, the moderation findings were interpreted with appropriate caution.

Overall, the structural model provided support for 9 of the 13 hypothesized relationships.

### Moderation analysis

Moderation analysis indicated significant digital literacy moderation effects for both hypothesized relationships involving net benefits. Digital literacy positively moderated the association between use and net benefits (*β* = 0.149, *p* = .031), indicating that the positive association between use and net benefits was stronger at higher levels of digital literacy. Conversely, digital literacy negatively moderated the association between user satisfaction and net benefits (*β* = −0.158, *p* = .019), indicating that the positive association between user satisfaction and net benefits was weaker at higher levels of digital literacy.

Simple slope analyses further illustrated these interaction patterns. As shown in Figure 3, the positive association between use and net benefits became stronger with increasing levels of digital literacy. In contrast, Figure 4 shows that the positive association between user satisfaction and net benefits became less pronounced as digital literacy increased.

**Figure 3:**
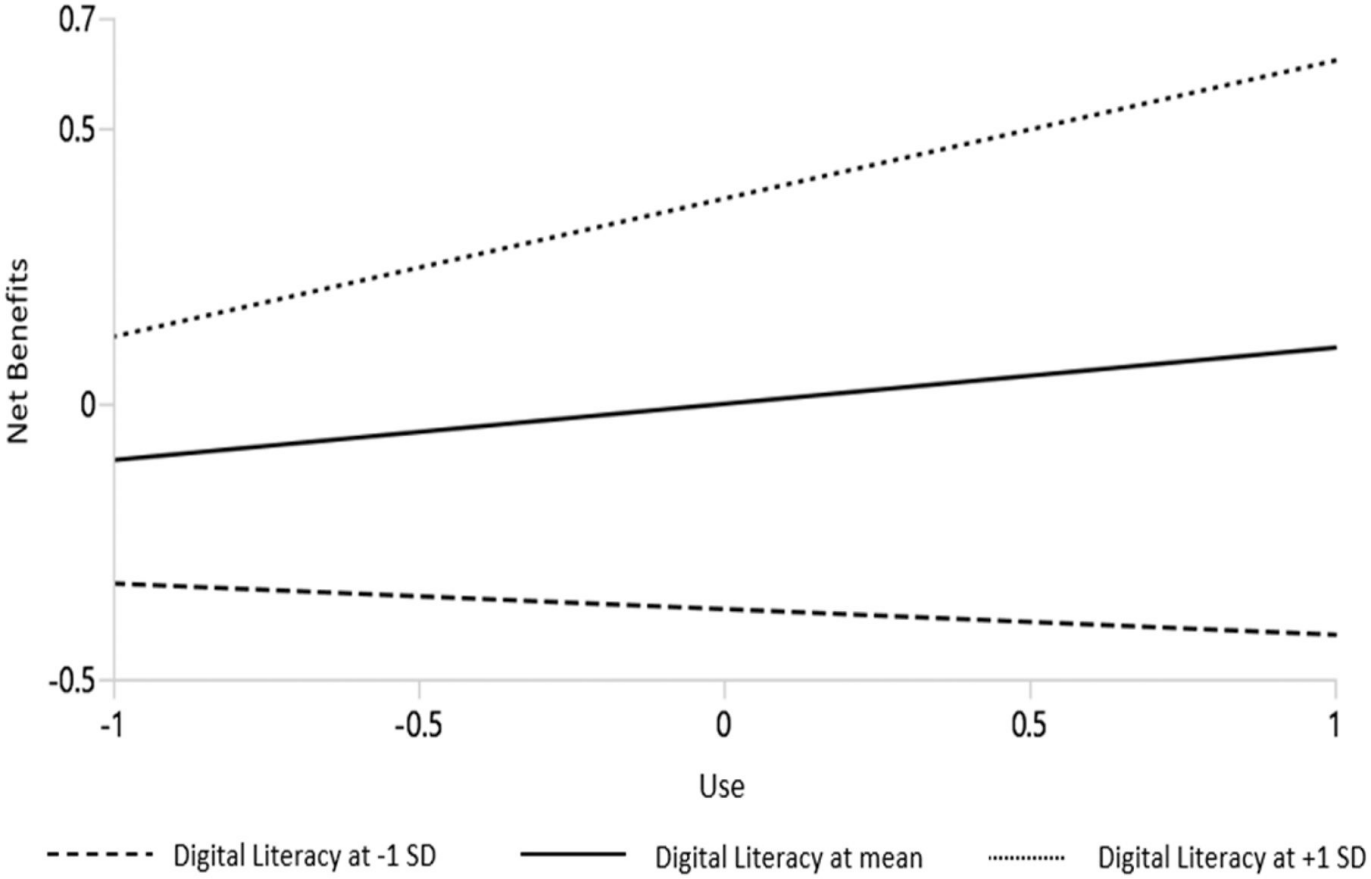
Moderating effect of digital literacy on the association between use and net benefits. Simple slope estimates are shown for low (−1 standard deviation [SD]), mean, and high (+1 SD) levels of digital literacy.

**Figure 4:**
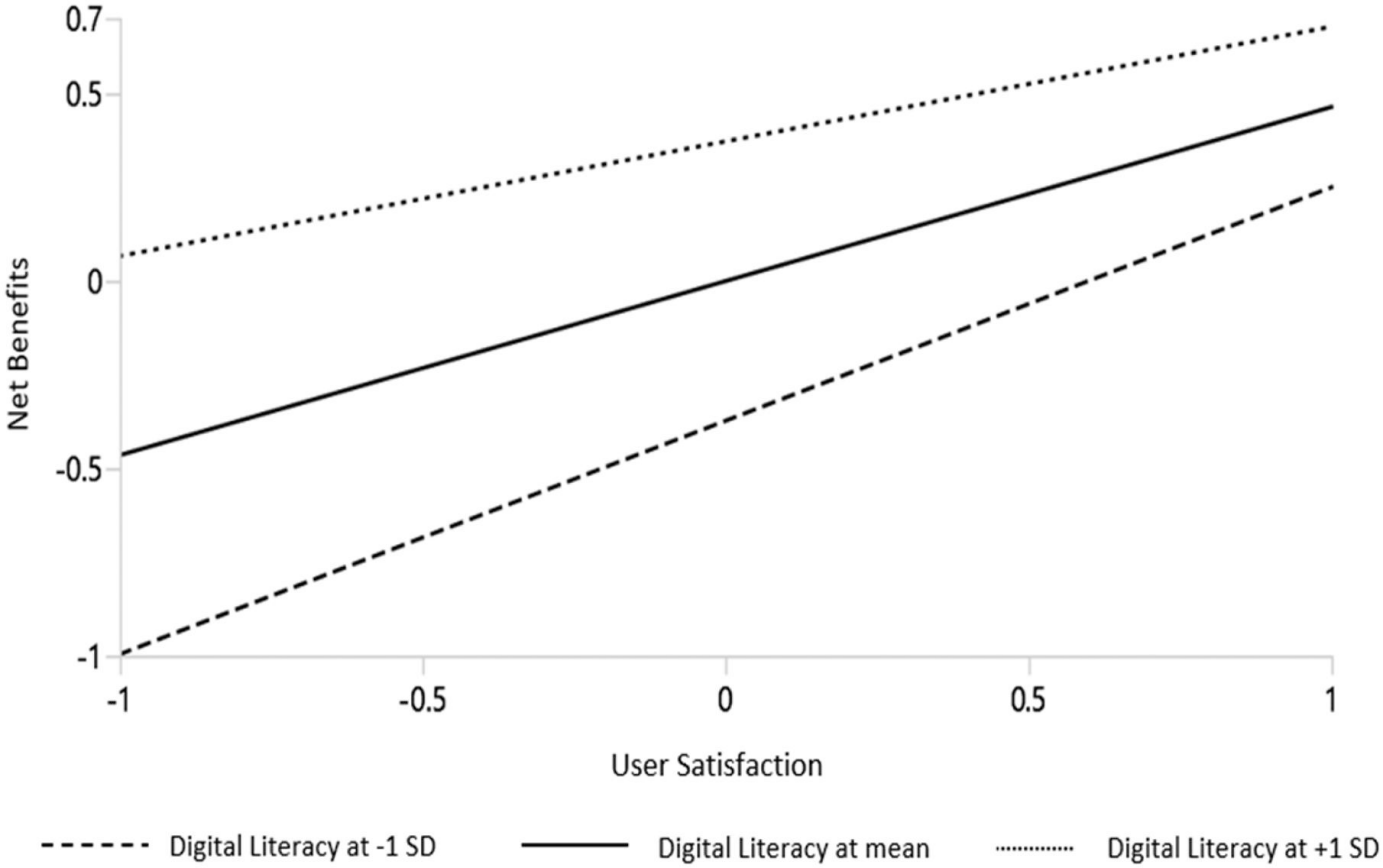
Moderating effect of digital literacy on the association between user satisfaction and net benefits. Simple slope estimates are shown for low (−1 standard deviation [SD]), mean, and high (+1 SD) levels of digital literacy.

### Importance–performance map analysis

Based on the structural model results, user satisfaction was selected as the target construct for the importance–performance map analysis (IPMA).

The IPMA identified AI explainability (HAI2), adaptive personalization (HAI3), system stability (SYS1), and functional completeness (SYS2) as indicators with high importance but comparatively lower performance, indicating substantial potential for improvement. In contrast, safeguards for personal information (HAI4), AI supporting rather than replacing human judgment (HAI1), and interface clarity (SYS3) demonstrated both high importance and high performance. Information quality indicators (INF1–INF3) generally exhibited high performance but comparatively lower importance, whereas service quality indicators (SER1–SER4) showed both lower importance and lower performance with respect to user satisfaction.

The complete IPMA results are presented in Figure 5.

**Figure 5:**
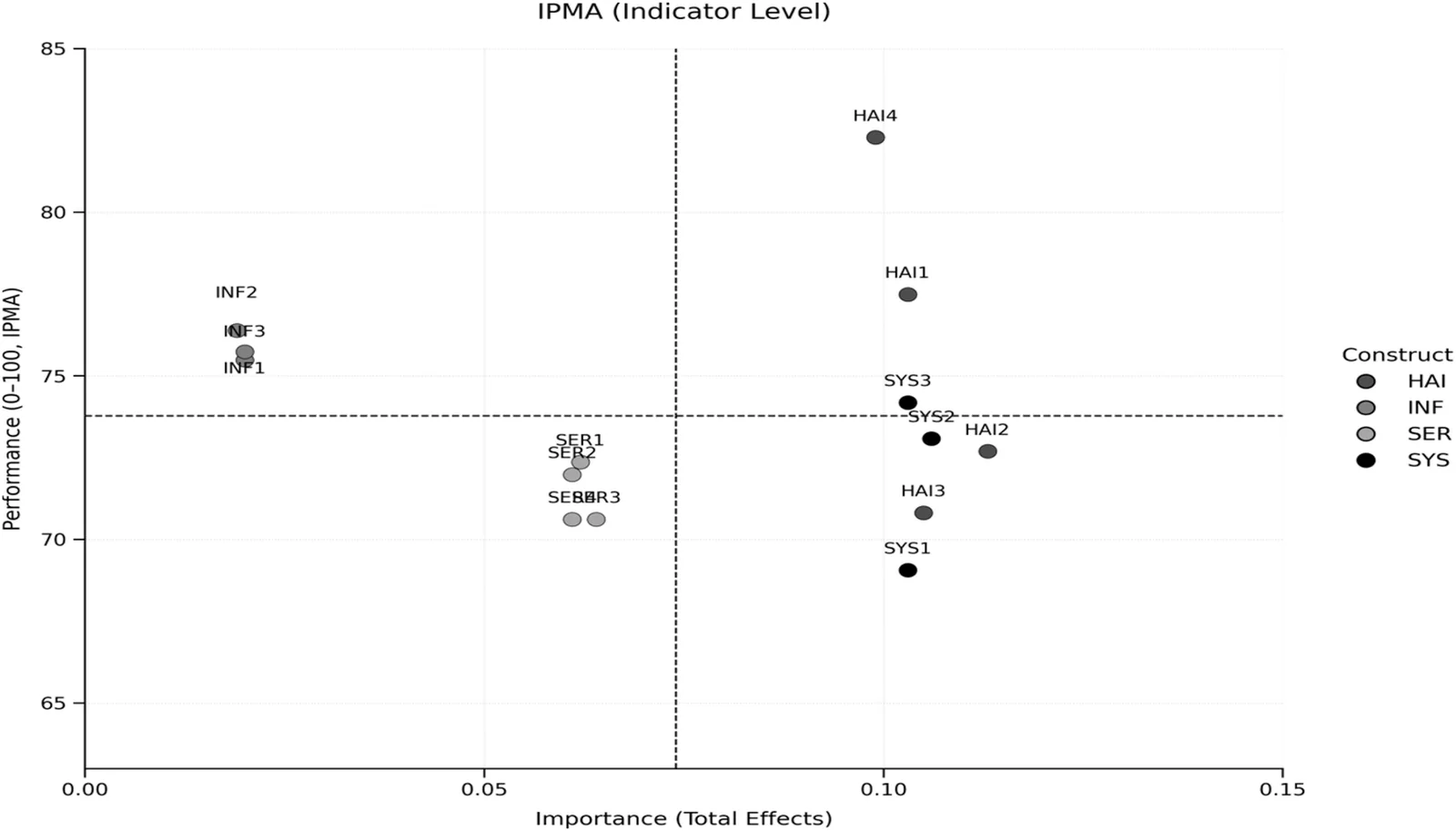
Importance–performance map analysis (IPMA) of user satisfaction. The figure displays the relative importance and performance of individual measurement items with respect to user satisfaction. Measurement items correspond to the constructs presented in Table 1. Abbreviations: IPMA, importance-performance map analysis; HAI, human-AI collaboration; INF, information quality; SER, service quality; SYS, system quality.

## Discussion

### Principal findings

This study investigated how organizational value is realized from AI-enabled digital health technologies (DHTs) in long-term care (LTC) settings. Within the framework examined in this study, organizational value was operationalized as respondents’ perceptions of value across care quality, operational efficiency, resource utilization, and workforce capability development. The findings indicate that perceived organizational value is associated with user experiences, human–AI collaboration, and workforce capability rather than technical quality or system use alone. Together, these findings suggest a value realization process through which AI-enabled DHTs may contribute to healthcare delivery optimization in LTC organizations.

Drawing on evidence from multiple LTC facilities, user satisfaction appeared to play a more prominent role than system use in explaining organizational value. Although system use was associated with several upstream quality dimensions, its direct association with net benefits was not statistically significant. In contrast, user satisfaction demonstrated a strong positive association with both system use and net benefits. These findings suggest that, in regulated care environments where technology use is largely mandatory, users’ experiences may provide a more meaningful indicator of information systems success than usage intensity alone. This interpretation is consistent with the meaningful use perspective in health information systems, which emphasizes quality, efficiency, and care improvement rather than simple utilization volume (61, 62). It also contrasts with much of the existing health information systems literature, which frequently reports more direct positive associations between system use or use intention and task-level outcomes (25, 63).

The findings further suggest that different quality dimensions play distinct roles in the value realization process. System quality was primarily associated with user satisfaction, whereas information quality was primarily associated with system use. Given the substantial empirical overlap between system quality and service quality, their construct-specific effects should nevertheless be interpreted with appropriate caution. Service quality demonstrated a negative association with system use. One possible explanation is that greater support needs may arise during periods of implementation difficulty, system complexity, or troubleshooting. However, alternative explanations, including conceptual overlap among quality constructs and the possibility of reverse or reciprocal relationships, cannot be excluded given the cross-sectional design. This unexpected finding should therefore be interpreted cautiously and warrants further investigation using longitudinal designs.

One of the most important findings of this study was that human–AI collaboration quality emerged as an additional dimension associated with information systems success in AI-enabled care environments, demonstrating significant associations with both system use and user satisfaction. This finding is consistent with growing evidence that successful AI implementation in healthcare depends not only on algorithmic performance but also on effective collaboration between healthcare professionals and AI systems (14–16, 18). Characteristics represented within this construct—including explainability, adaptive personalization, support for human judgment, and safeguards for personal information—appear particularly relevant for facilitating users’ engagement with AI-supported care processes and shaping their overall experiences with AI-enabled DHTs.

The study also identified significant moderation effects associated with digital literacy. Digital literacy strengthened the relationship between system use and net benefits while attenuating the relationship between user satisfaction and net benefits. Higher levels of digital literacy strengthened the extent to which system use was associated with net benefits, suggesting that digitally literate users may be better able to translate system engagement into organizational outcomes. This interpretation is consistent with prior research identifying digital literacy as a key workforce capability that supports effective engagement with digital health technologies and successful digital transformation in healthcare (22, 23, 46). At the same time, the weaker association between user satisfaction and net benefits among users with higher digital literacy may reflect contextual characteristics of LTC environments, differences in user expectations, or other factors not captured in the present study. The interpretation of this moderation pattern should therefore be considered exploratory. Given the greater methodological complexity and statistical demands associated with moderation analysis, these findings should be interpreted with appropriate caution and further examined in larger samples.

Collectively, these findings suggest that organizational value realization from AI-enabled DHTs is best understood as a socio-technical process in which technical system characteristics, human–AI collaboration, user experiences, and workforce capability are interrelated with perceived organizational value. This interpretation aligns with growing recognition that human-centered AI design and effective human–AI collaboration play central roles in shaping the impact of AI-enabled DHTs in healthcare (16, 18, 65).

Complementing these findings, the IPMA results identified AI explainability, adaptive personalization, system stability, and functional completeness as the highest-priority targets for improvement. In contrast, safeguards for personal information, support for human judgment, and interface clarity emerged as relatively strong-performing areas that should be maintained. Together, these findings identify system and human–AI collaboration characteristics that may be particularly important for supporting organizational value realization in LTC settings.

### Theoretical implications

This study contributes to the literature of AI-enabled digital health and information systems by extending current understanding of how organizational value is realized from AI-enabled digital health technologies (DHTs) in long-term care (LTC) settings. Building on the DeLone and McLean (D&M) Information Systems Success Model, the findings suggest that realizing organizational value from AI-enabled DHTs involves not only traditional technical quality dimensions but also collaborative human–AI interactions and workforce capability. Together, these findings broaden existing perspectives on information systems success in AI-enabled healthcare environments.

First, this study extends the D&M model by suggesting that organizational value in AI-enabled care environments may not be adequately explained through system utilization alone. Although the original model conceptualizes both system use and user satisfaction as important antecedents of net benefits, the present findings indicate that user satisfaction was more strongly associated with organizational value than system use. These findings reinforce the importance of experiential and outcome-oriented measures alongside utilization indicators when evaluating information systems success in AI-enabled care environments.

Second, this study extends the Information Systems Success Model by conceptualizing human–AI collaboration quality as a distinct interactional quality construct that complements the established information, system, and service quality dimensions for evaluating AI-enabled DHTs. Traditional information systems success models primarily emphasize information quality, system quality, and service quality as key determinants of system success (12, 13). However, AI-enabled DHTs increasingly involve collaborative interactions between healthcare professionals and AI systems that are not fully captured by these traditional quality dimensions (16, 18, 64). The proposed construct broadens existing information systems evaluation frameworks by explicitly incorporating collaborative and human-centered characteristics—including explainability, support for human judgment, adaptive personalization, and safeguards for personal information—into the evaluation of AI-enabled DHTs (12, 13, 65, 66).

Third, the findings broaden current understanding of digital literacy by suggesting that its theoretical role extends beyond facilitating technology adoption and effective system use. Previous research has largely conceptualized digital literacy as an individual competency supporting engagement with digital technologies (22, 23, 45). The present findings suggest that digital literacy may also function as a boundary condition by moderating the associations of system use and user satisfaction with organizational value, thereby extending the theoretical role of workforce capability within information systems evaluation.

Finally, this study contributes to the relatively limited body of evidence examining AI-enabled digital health technologies in long-term care settings. Much of the existing literature has focused on hospital environments, frequently relying on single-institution samples. By examining multiple LTC facilities with diverse workforce characteristics, the present study provides evidence from an underexplored care context and suggests that value realization processes may differ across healthcare environments.

Collectively, these theoretical contributions suggest that understanding organizational value realization from AI-enabled DHTs requires moving beyond traditional evaluations centered primarily on technical system characteristics and technology use. Extending information systems success theory to AI-enabled healthcare environments requires explicit consideration of collaborative human–AI interactions and workforce capability alongside established quality dimensions. In doing so, this study provides a more comprehensive theoretical framework for understanding how organizational value is realized from AI-enabled DHTs in LTC settings and offers a foundation for future research on AI-enabled healthcare management in aging societies.

### Practical implications

The findings provide practical implications for long-term care (LTC) organizations seeking to realize organizational value from AI-enabled digital health technologies (DHTs) in the context of population aging, increasing care complexity, and workforce shortages. AI-enabled DHTs should not be viewed solely as technical solutions for care delivery. The findings suggest that realizing organizational value is associated with both technological and human factors, including effective human–AI collaboration, workforce digital capability, and user satisfaction. Successful implementation may therefore require coordinated attention to system design, workforce development, and organizational management strategies that support data-driven approaches to optimizing healthcare delivery in LTC settings.

#### Human-centered system design

The findings suggest that organizations should prioritize human-centered approaches when developing, selecting, and implementing AI-enabled DHTs. Human–AI collaboration quality was significantly associated with both system use and user satisfaction, suggesting that successful implementation may involve not only technical performance but also effective support for collaboration between users and AI systems. AI systems that incorporate explainability, adaptive personalization, and safeguards for personal information may help users better interpret and apply AI-generated information to support care-related decision-making in routine practice. These principles are consistent with recommendations from the human-centered AI and explainable AI literature, which emphasize transparency, trustworthiness, and user-centered design as important factors supporting the effective implementation of AI-enabled DHTs in healthcare settings (14, 65–67).

The importance-performance map analysis further identified AI explainability, adaptive personalization, system stability, and functional completeness as priority areas for improvement. These findings suggest that organizations and technology developers should prioritize these characteristics when implementing or refining AI systems within AI-enabled DHTs to enhance user satisfaction and support organizational value realization.

#### Workforce digital capability

The findings further suggest that workforce digital capability is an important organizational resource for realizing value from AI-enabled DHTs. The significant moderation effects of digital literacy highlight the importance of workforce readiness as part of digital transformation efforts in LTC settings. Accordingly, LTC organizations should consider digital literacy development as an ongoing organizational investment rather than a one-time implementation activity. Targeted training programs, continuous learning opportunities, and practical user support may strengthen users’ ability to engage effectively with AI-enabled DHTs and integrate digital technologies into routine care delivery. Given the diversity of LTC workforces, implementation strategies should be tailored to varying levels of digital proficiency and organizational readiness (46, 68).

#### Organizational strategies and digital transformation

For LTC leaders and managers, implementation efforts should extend beyond technology acquisition and deployment to include workforce engagement, workflow integration, and ongoing evaluation of organizational outcomes (10). Although technology utilization remains important, the findings suggest that implementation success should be evaluated using broader indicators of organizational value rather than system use alone. Consistent with the dimensions of organizational value examined in this study, organizations may benefit from assessing whether AI-enabled DHTs contribute to improvements in care quality, operational efficiency, resource utilization, and workforce capability development. Monitoring these organizational outcomes may help LTC leaders evaluate the value generated by digital transformation initiatives and support data-driven strategies for optimizing healthcare delivery and sustainable long-term care in aging societies.

### Limitations and future research

This study has several limitations. First, the cross-sectional design and reliance on self-reported data limit the ability to establish temporal ordering or causal relationships. Accordingly, the observed relationships should be interpreted as associations rather than causal effects. The design also precluded examination of potentially reciprocal relationships between system use and user satisfaction over time (13, 43) and the evolving role of trust in shaping these user responses. The use of a single questionnaire may also increase the potential for common-method bias. Future research may adopt longitudinal or quasi-experimental designs to examine these dynamic relationships and incorporate objective indicators, such as system logs and organizational outcome measures, to strengthen causal inference and further validate the observed relationships.

Second, the use of convenience sampling limits the extent to which the sample can be considered representative of Taiwan's broader LTC workforce. Nevertheless, the sample showed broad similarity to available year-end 2025 national workforce statistics for elderly long-term care, nursing, and caring institutions in some observable characteristics (69). Although participants were recruited from multiple LTC facilities, possible within-facility clustering was not accounted for in the analysis. Future studies using broader and more diverse samples could further examine the robustness and generalizability of the findings.

Third, the study was conducted in LTC settings in Taiwan, which may limit generalizability to other healthcare and cultural contexts. The heterogeneity of AI-enabled DHTs included in the study may also have introduced unobserved variation across technology and implementation contexts, including differences in implementation maturity and duration of sustained technology use. Differences in workflows and technology-use contexts across LTC service settings may represent an additional source of contextual variation and warrant further examination in larger setting-specific samples. In addition, the human–AI collaboration quality measure represents an initial operationalization developed for this study and warrants further validation across independent samples, healthcare settings, and cultural environments.

## Conclusions

This study examined factors associated with organizational value realization from AI-enabled digital health technologies (DHTs) in long-term care (LTC) settings by extending the DeLone and McLean Information Systems Success Model to incorporate human–AI collaboration quality and digital literacy. The findings suggest that realizing organizational value from AI-enabled DHTs involves more than traditional technical quality dimensions and system use alone. A human-centered approach that prioritizes explainability, adaptive personalization, system stability, and functional completeness, while strengthening workforce digital capability, may help support organizational value realization. Within the framework examined in this study, organizational value was reflected in perceived improvements in care quality, operational efficiency, resource utilization, and workforce capability development. Together, these dimensions of perceived organizational value provide a practical pathway through which AI-enabled DHTs may support healthcare delivery optimization and the long-term sustainability of LTC services.

As LTC systems face increasing pressures associated with population aging, workforce shortages, and growing care demands, organizations are increasingly seeking ways to optimize healthcare delivery through digital innovation. The findings provide insights into how organizational value is realized from AI-enabled DHTs in LTC settings and offer a framework for understanding the interconnected roles of human-centered system design, user experiences, workforce digital capability, and organizational outcomes. The study further offers practical guidance for LTC managers regarding the implementation and evaluation of AI-enabled DHTs, emphasizing the importance of aligning technology investments with strategic organizational objectives and longer-term organizational value. Together, these findings advance current understanding of organizational value realization from AI-enabled DHTs and provide a foundation for supporting more effective and sustainable long-term care in aging societies.

## Data Availability

The datasets generated and/or analyzed during the current study are not available due to institutional and ethical restrictions.
