# Supplementary material for: Organizational value realization from AI-enabled digital health technologies in long-term care: roles of human–AI collaboration and digital literacy
Source: Front Med (Lausanne). 2026 Oct 14;13:1958359. doi: 10.3389/fmed.2026.1958359 (PMC13713272; doi:10.3389/fmed.2026.1958359)
Supplement: Supplementary file 1 [file Datasheet1.pdf]

C114027

超高齡社會下長照機構導入智慧科技系統的關鍵成功因素：從長照人員視角出發

親愛的長照工作夥伴您好：

感謝您撥冗閱讀本研究的說明，配合此次的問卷調查。您的參與對我們而言意義深遠。

本問卷採匿名方式進行，所蒐集之資料僅供學術研究分析使用。若您在填答過程中有任何疑問，可隨時中止填寫，或與研究人員蔡玲玉聯繫。

為保障您的權益並確保資料品質，問卷首頁將提供「系統」相關名詞定義，並再次確認您的參與資格。勾選「我已閱讀本研究的說明並同意參與研究」後，即可開始填寫問卷。問卷共35題選擇題，最後一頁請留下手機號碼或電子郵箱，以便寄送電子禮卡。

衷心感謝您的支持與協助，讓我們共同為提升長期照護的品質與效率盡一份心力。

敬祝

平安順心、萬事如意

指導教授：陳銘芷、藍易振博士
博士候選人：蔡玲玉
聯絡方式：0958-258-078 或 grace.ly.tsai@hotmail.com
中華民國一一四年九月

* Indicates required question

「系統」名詞定義

問卷中所稱之「系統」乃「智慧科技」系統設備之簡稱，涵蓋範圍請參見示意圖如下。

核准章
114. 9. 09
輔仁大學
人體研究倫理委員會
請依核准版本執行

本研究所指的「智慧科技系統」範疇

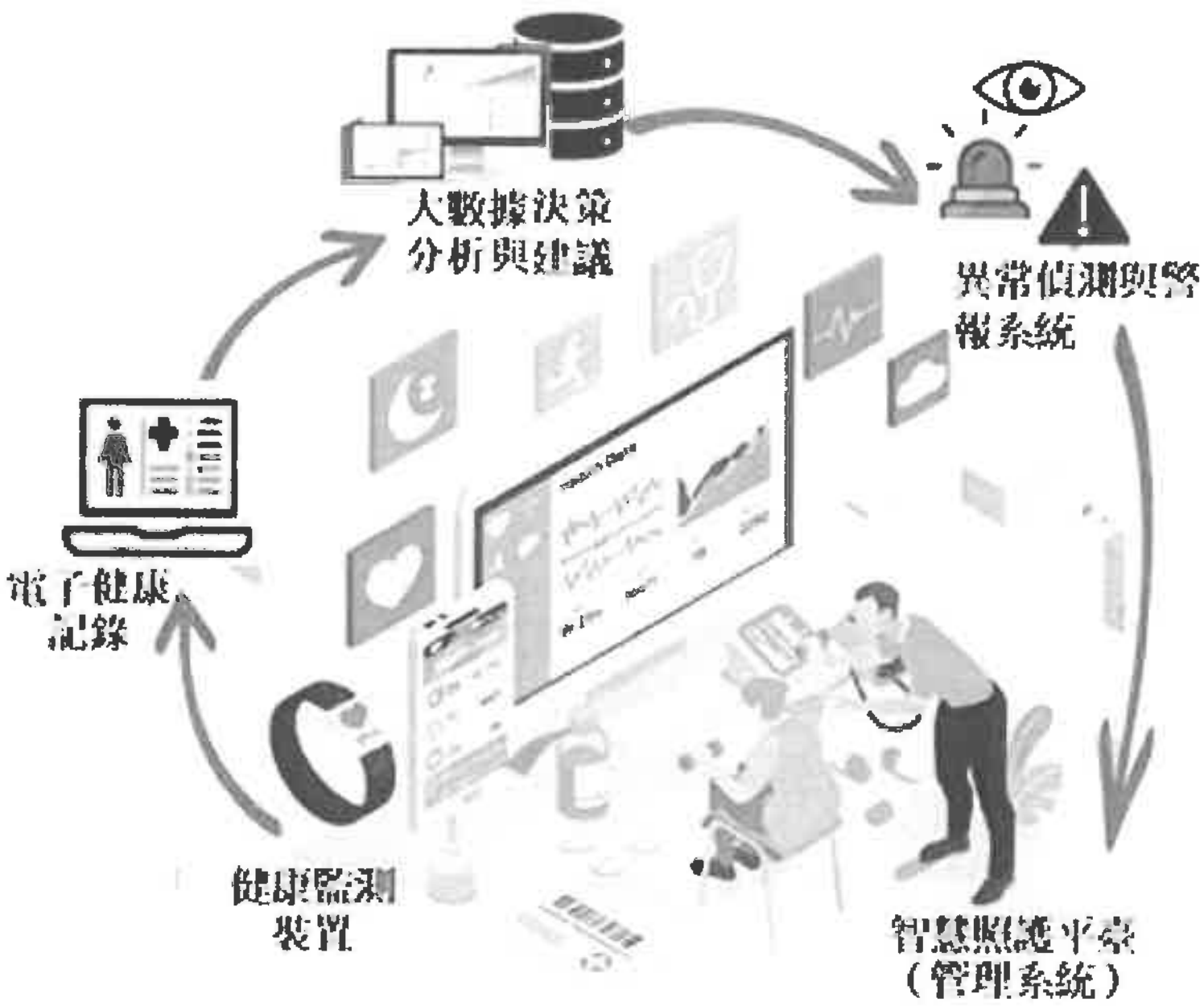

1. 您是否已閱讀本研究的說明，並同意參與研究？ *

Mark only one oval.

- ( ) 是，我同意
- ( ) 否，我不同意（若選擇此項，請勿繼續填寫）
  *Skip to section 4 (**問卷結束**（未同意參與))*

參與資格

2. 您目前是否任職於長照機構，且具備6個月以上的系統使用經驗（包含過去工作經歷）？（必填） *

Mark only one oval.

- ( ) 是的 *Skip to question 3*
- ( ) 不是 *Skip to section 5 (**問卷結束**(非任職於長照機構，或使用系統不到6個月))*

問卷結束（未同意參與)

感謝您點閱本問卷。
由於您選擇不同意參與本研究，我們將不收集您的任何資料，問卷至此結束。

若您日後改變想法，歡迎研究團隊聯繫。

祝您一切順心，謝謝！

Skip to question 3

問卷結束（ 非任職於長照機構，或使用系統不到6個月)

感謝您填寫本表單。
根據您的回覆，您目前未任職於長照機構，或尚未累積六個月以上的智慧科技系統使用經驗。
因此，您的資料將不納入本研究此階段的分析。

非常感謝您的參與與支持，未來若有相關研究或後續調查，歡迎您再次參與！

參與人員基本資料

3. **年齡：** *

Mark only one oval.

- 30歲以下
- 31–40歲
- 41–50歲
- 51–60歲
- 60歲以上

4. **性別：** *

Mark only one oval.

- 女
- 男
- 其他

5. **最高學歷：** *

Mark only one oval.

- 高中（職）或以下
- 專科
- 大學
- 碩士
- 博士

6. **年資（在目前機構工作年資）：** *

Mark only one oval.

- 6個月及以下
- 6個月以上至12個月
- 1年以上至3年
- 3年以上至10年
- 10年以上

7. **服務機構類型：** *

Mark only one oval.

- 居家服務單位
- 日間照顧中心
- 住宿型照顧機構
- 其他

8. **職位：(可複選)** *

Check all that apply.

- 照顧服務員
- 護理師
- 社工師
- 主任
- 督導
- 業務負責人
- 其他

系統的技術品質

由系統產生的資訊、操作功能與技術支援服務品質，包括準確、有用、易用、可靠、與即時性。

9. 系統記錄和提供符合照護工作需要的資訊。 *

Mark only one oval.

	1	2	3	4	5	6	7	
非常								非常同意

10. 系統提供的訊息或內容是正確的。 *

Mark only one oval.

	1	2	3	4	5	6	7	
非常	◯	◯	◯	◯	◯	◯	◯	非常同意

11. 系統提供的資訊清楚易懂。 *

Mark only one oval.

	1	2	3	4	5	6	7	
非常	◯	◯	◯	◯	◯	◯	◯	非常同意

12. 系統運作很穩定。 *

Mark only one oval.

	1	2	3	4	5	6	7	
非常	◯	◯	◯	◯	◯	◯	◯	非常同意

13. 系統功能齊全，對工作很有幫助。 *

Mark only one oval.

	1	2	3	4	5	6	7	
非常	◯	◯	◯	◯	◯	◯	◯	非常同意

14. 系統的操作介面設計清晰合理，容易上手。*

Mark only one oval.

	1	2	3	4	5	6	7	
非常	◯	◯	◯	◯	◯	◯	◯	非常同意

15. 系統人員的服務是可靠的。*

Mark only one oval.

	1	2	3	4	5	6	7	
非常	◯	◯	◯	◯	◯	◯	◯	非常同意

16. 系統人員能理解我的需求。*

Mark only one oval.

	1	2	3	4	5	6	7	
非常	◯	◯	◯	◯	◯	◯	◯	非常同意

17. 系統人員能及時回應我的問題。*

Mark only one oval.

	1	2	3	4	5	6	7	
非常	◯	◯	◯	◯	◯	◯	◯	非常同意

18. 系統人員提供足夠的使用說明與訓練。 *

Mark only one oval.

	1	2	3	4	5	6	7	
非常	○	○	○	○	○	○	○	非常同意

系統的人本AI協作品質

指系統設計是否以「人」為核心，使用者與人工智慧之間的互補與協作關係。此品質主要衡量系統給予使用者自主性，可解釋性、參與感與安全感的程度。

19. 系統可輔助我的工作，非取代我的判讀或決定。 *

Mark only one oval.

	1	2	3	4	5	6	7	
非常	○	○	○	○	○	○	○	非常同意

20. 系統提供的建議或預測邏輯容易理解。 *

Mark only one oval.

	1	2	3	4	5	6	7	
非常	○	○	○	○	○	○	○	非常同意

21. 系統可根據使用者的需要或反饋，調整訊息或內容呈現的方式。 *

Mark only one oval.

	1	2	3	4	5	6	7	
非常	○	○	○	○	○	○	○	非常同意

22. 系統有密碼與權限設置等保護個人資料的機制，避免外洩。 *

Mark only one oval.

	1	2	3	4	5	6	7	
非常	◯	◯	◯	◯	◯	◯	◯	非常同意

系統使用意願與滿意度

23. 我經常使用系統來完成日常工作。 *

Mark only one oval.

	1	2	3	4	5	6	7	
非常	◯	◯	◯	◯	◯	◯	◯	非常同意

24. 我覺得這個系統對我完成工作非常重要。 *

Mark only one oval.

	1	2	3	4	5	6	7	
非常	◯	◯	◯	◯	◯	◯	◯	非常同意

25. 我在需要完成任務時會主動使用這個系統。 *

Mark only one oval.

	1	2	3	4	5	6	7	
非常	◯	◯	◯	◯	◯	◯	◯	非常同意

26. 我對系統的整體服務感到滿意。 *

Mark only one oval.

	1	2	3	4	5	6	7	
非常	◯	◯	◯	◯	◯	◯	◯	非常同意

27. 我的系統使用經驗是愉悅的。 *

Mark only one oval.

	1	2	3	4	5	6	7	
非常	◯	◯	◯	◯	◯	◯	◯	非常同意

28. 我願意推薦此系統給其他人使用 。 *

Mark only one oval.

	1	2	3	4	5	6	7	
非常	◯	◯	◯	◯	◯	◯	◯	非常同意

數位素養

指系統使用者在工作日常中，能夠自信且具批判性地運用智慧科技工具與系統，進行資訊的查詢、溝通與應用的能力。

29. 我能根據系統顯示的訊息或資訊，採取適當的照護行動或工作決策。 *

Mark only one oval.

	1	2	3	4	5	6	7	
非常	◯	◯	◯	◯	◯	◯	◯	非常同意

30. 我能使用數位（如Line，電子郵件等) 平台，與工作夥伴或系統人員清楚溝通。 *

Mark only one oval.

	1	2	3	4	5	6	7	
非常	◯	◯	◯	◯	◯	◯	◯	非常同意

31. 我能在系統中撰寫與照護相關的紀錄、回饋或建議。 *

Mark only one oval.

	1	2	3	4	5	6	7	
非常	◯	◯	◯	◯	◯	◯	◯	非常同意

32. 當系統操作遇到困難時，我能嘗試自行解決或尋求協助 。 *

Mark only one oval.

	1	2	3	4	5	6	7	
非常	◯	◯	◯	◯	◯	◯	◯	非常同意

系統使用效益

指智慧科技系統在實際應用中，對提升服務品質、節省人力與資源、強化作業效率，以及促進專業技能發展的貢獻程度。

33. 系統幫助提升服務對象的安全與照護品質。 *

Mark only one oval

	1	2	3	4	5	6	7	
非常	◯	◯	◯	◯	◯	◯	◯	非常同意

34. 系統節省長照機構資源（如紙張、水電、醫療用品、人力）。*

Mark only one oval.

	1	2	3	4	5	6	7	
非常	○	○	○	○	○	○	○	非常同意

35. 系統提升服務工作的效率。*

Mark only one oval.

	1	2	3	4	5	6	7	
非常	○	○	○	○	○	○	○	非常同意

36. 系統培訓提升我的學習效率與專業技能。*

Mark only one oval.

	1	2	3	4	5	6	7	
非常	○	○	○	○	○	○	○	非常同意

37. 除了問卷中的選項外，您覺得還有哪些系統功能或品質能提升工作效率或照護品質？請選出最重要的三項： *

Check all that apply.

- [ ] 與行政醫療系統整合
- [ ] 資料查詢方便
- [ ] 自動紀錄功能
- [ ] 操作介面簡單好用
- [ ] 管理報表輔助決策
- [ ] 內建學習資源
- [ ] 個案風險預警分析
- [ ] 提供個人化照護建議
- [ ] 其它

酬謝禮卡寄送方式

為寄送酬謝禮卡，請您提供手機號碼或電子郵件（擇一填寫即可）。

此資料僅用於寄送用途，不會用於其他用途。

38. 請確認輸入正確的台灣手機號碼（共 10 位數，例如：0912345678）或電子郵箱（例如：x.x@xx.com）。 若您選擇提供電子郵箱，請留意信件是否被誤判為垃圾郵件。

您的寶貴意見對我們深入了解長照機構導入智慧科技系統的關鍵成功因素具有重要意義。

若您有任何疑問或建議，歡迎透過電子郵件（grace.ly.tsai@hotmail.com）與我們聯繫。

再次感謝您的支持與參與，祝您平安順心、萬事如意！
版本：3
版本日期：2025/9/3

This content is neither created nor endorsed by Google.

Google Forms
