# Supplementary material for: Organizational value realization from AI-enabled digital health technologies in long-term care: roles of human–AI collaboration and digital literacy
Source: Front Med (Lausanne). 2026 Oct 14;13:1958359. doi: 10.3389/fmed.2026.1958359 (PMC13713272; doi:10.3389/fmed.2026.1958359)

Supplementary Figure S1. Participant-facing illustration defining the scope of AI-enabled digital health technologies (DHTs) in long-term care. English-translated version of the original Chinese illustration presented to participants at the commencement of the questionnaire.

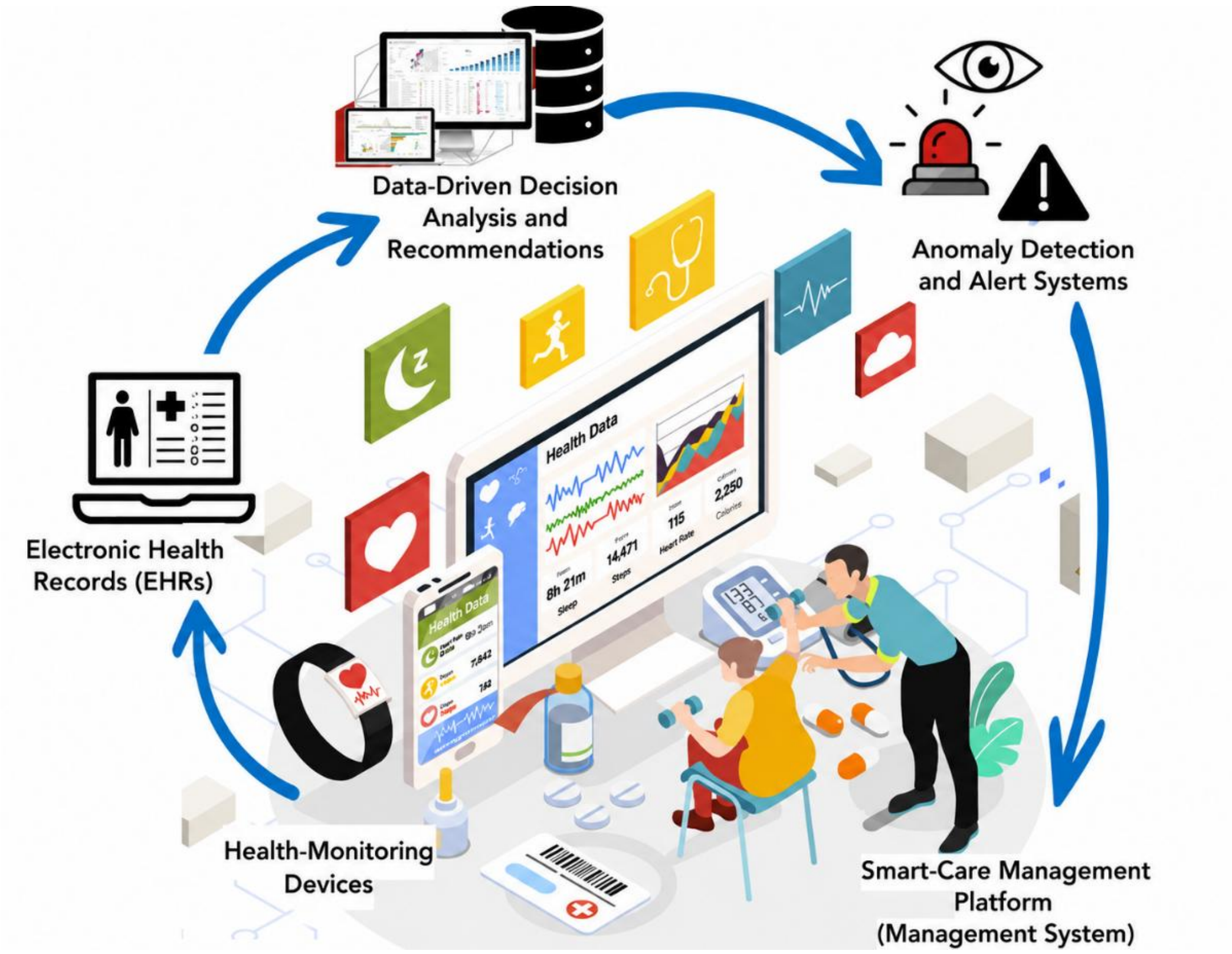

Supplement: Supplementary file 2 [file Datasheet2.pdf]
